# Subtype-Based Prognostic Analysis of Cell-in-Cell Structures in Early Breast Cancer

**DOI:** 10.3389/fonc.2019.00895

**Published:** 2019-09-20

**Authors:** Xin Zhang, Zubiao Niu, Hongquan Qin, Jie Fan, Manna Wang, Bo Zhang, You Zheng, Lihua Gao, Zhaolie Chen, Yanhong Tai, Mo Yang, Hongyan Huang, Qiang Sun

**Affiliations:** ^1^Department of Pediatric, Nanfang Hospital, Southern Medical University, Guangzhou, China; ^2^Laboratory of Cell Engineering, Institute of Biotechnology, Beijing, China; ^3^The Seventh Affiliated Hospital, Sun Yat-sen University, Guangzhou, China; ^4^Department of Oncology, Beijing Shijitan Hospital of Capital Medical University, Beijing, China; ^5^Department of Pathology, The Fifth Medical Center, General Hospital of PLA, Beijing, China; ^6^Lian Jiang People's Hospital, Lianjiang, China

**Keywords:** breast cancer, cell-in-cell structures, macrophage, overall survival, prognosis

## Abstract

Though current pathological methods are greatly improved, they provide rather limited functional information. Cell-in-cell structures (CICs), arising from active cell–cell interaction, are functional surrogates of complicated cell behaviors within heterogeneous cancers. In light of this, we performed the subtype-based CIC profiling in human breast cancers by the “EML” multiplex staining method, and accessed their values as prognostic factors by Cox univariate, multivariate, and nomogram analysis. CICs were detected in cancer specimens but not in normal breast tissues. A total of five types of CICs were identified with one homotypic subtype (91%) and four heterotypic subtypes (9%). Overall CICs (oCICs) significantly associated with patient overall survival (OS) (*P* = 0.011) as an independent protective factor (HR = 0.423, 95% CI, 0.227–0.785; *P* = 0.006). Remarkably, three CICs subtypes (TiT, TiM, and MiT) were also independent prognostic factors. Among them, higher TiT, from homotypic cannibalism between tumor cells, predicted longer patient survival (HR = 0.529, 95% CI, 0.288–0.973; *P* = 0.04) in a way similar to that of oCICs and that (HR = 0.524, 95% CI, 0.286–0.962; *P* = 0.037) of heterotypic TiM (tumor cell inside macrophage); conversely, the presence of MiT (macrophage inside tumor cell) predicted a death hazard of 2.608 (95% CI, 1.344–5.063; *P* = 0.05). Moreover, each CIC subtype tended to preferentially affect different categories of breast cancer, with TiT (*P* < 0.0001) and oCICs (*P* = 0.008) targeting luminal B (Her2^+^), TiM (*P* = 0.011) targeting HR^−^ (Her2^+^/HR^−^ and TNBC), and MiT targeting luminal A (*P* = 0.017) and luminal B (Her^−^) (*P* = 0.006). Furthermore, nomogram analysis suggested that CICs impacted patient outcomes in contributions comparable (for oCICs, TiT, and TiM), or even superior (for MiT), to TNM stage and breast cancer subtype, and incorporating CICs improved nomogram performance. Together, we propose CICs profiling as a valuable way for prognostic analysis of breast cancer and that CICs and their subtypes, such as MiT, may serve as a type of novel functional markers assisting clinical practices.

## Introduction

Breast cancer, like all the other types of human tumors, is highly heterogeneous at both molecular and cellular levels (1, 2) and therefore requires careful subtyping prior to further clinical managements. Though the subtypes based on traditional pathology and receptor statuses are well-accepted to stratify breast cancer patients, patients within each subtype remain variable in terms of recurrence, mortality rates, and prognosis (3, 4). Whereas, recently developed molecular profiling is promising in classifying patients based on sets of specified genes, the method, like traditional pathology, provided rather limited functional information (5–7). We sought to develop a functional way that reflects the phenotype of tumor malignancy within tumor microenvironment rather than changes in molecular or single cell levels.

Cell-in-cell structures (CICs) are unique in that morphologically intact cells stay inside another cell, which arise from active cell–cell interaction and are prevalent in a wide range of human cancers (8). Due to complex cellular composition in cancer tissues, CICs could form homotypically between tumor cells, or heterotypically between different types of cells, such as tumor cells and immune cells (9, 10). Formation of CICs generally leads to the death of internalized cells in a non-autonomous way, by which the inner cells were killed by the outer cells (11). Therefore, CIC-mediated cell death was proposed as the type IV cell death after apoptosis (type I), necrosis (type II), and autophagy-dependent cell death (type III) (12). CICs seem to play dual roles (suppressive and promotive) in human tumors, which are likely affected by multiple factors including CIC types, tumor types, stages, and so forth (8, 10). Hence, formation of a specified type of CICs is an integrative outcome of multiple signals (13–17) and may be used as a functional surrogate of tumor malignancy (18, 19). Consistently, accumulating data suggested that CICs were associated with tumor grades (19–21). Nevertheless, previous studies stained samples by H&E, or Giemsa, or simple immunohistochemistry etc., which could only detect unsubtyped CICs and read out limited functional information. Recently, we established a multiplex “EML” method (9) to subtype CICs in human cancer tissues, in which samples were simultaneously stained with antibodies against E-cadherin, CD68, and CD45, the markers for epithelial cancer cells, macrophages, and leukocytes, respectively. This method turned out to work well in a small amount of samples from different types of human cancers including breast cancer.

In this study, we employed CICs as a functional readout for complicated intracellular signaling and intercellular interactions, performed a systemic analysis of CIC subtypes by the established “EML” method, and accessed their values to assist prognostic and predictive decision-making in a cohort of human breast cancers.

## Materials and Methods

### Human Tumor Tissue Microarray

The human tumor tissue microarray (TMA) slide (HBre-Duc170Sur-01) from 170 patients with breast cancer was purchased from Shanghai Outdo Biotech Co. Ltd. A total of 170 cores on slide consisted of 160 cases of breast cancer tissue and 10 cases of normal breast tissue. The diameter of each core was 1.5 mm. For detailed information of each sample plotted on the slide, please visit the website of Shanghai Outdo (http://www.superchip.com.cn/biology/tissue). All tissues were collected under the highest ethical standards with the donor being informed completely and with their consent, from National Human Genetic Resources Sharing Service Platform: 2005DKA21300.

### Immunostaining and Image Processing

The “EML” method was used to subtype CICs as previously reported (9). In brief, samples were first stained with antibody against CD45 (mouse mAb from Boster, BM0091) at a dilution of 1:400 by Opal Multiplex tissue staining kit (Perkin Elmer, NEL791001KT) according to the standard protocol provided, and CD45 molecules were eventually labeled with Cyanine 5 fluorophore. Slides were then incubated with mixed antibodies against E-cadherin (mouse mAb from BD Biosciences, 610181) and CD68 (rabbit pAb from Proteintech, 25747–1-AP), followed by secondary antibodies of Alexa Fluor 568 anti-rabbit antibody (Invitrogen, A11036) and Alexa Fluor 488 anti-mouse antibody (Invitrogen, A11029). All slides were counterstained with DAPI to show nuclei and mounted with Antifade reagent (Invitrogen, Carlsbad, CA) and cover slips.

Multispectral images were taken with TMA modules of Vectra® Automated Imaging System (Perkin Elmer) by a 20× objective lens. Nuance system (Perkin Elmer) was used to build libraries of each spectrum (DAPI, FITC, TRITC, and Cy5) and unmix multispectral images with high contrast and accuracy. InForm automated image analysis software (Perkin Elmer) was used for batch analysis of multispectral images based on specified algorithms.

### Quantification of CICs in TMA

Cell-in-cell structures (CICs) were scored and quantified as previously described (9). Generally, CICs were first identified from a composite image with multiple fluorescent channels merged together and then confirmed in an individual fluorescent channel for CIC subtyping. The entire core (~1.76 mm^2^) of each tissue sample was screened except for blurred regions that were hard to read. A cellular structure where one or more cells are fully enclosed inside another cell with crescent nucleus was scored as CICs. Cell boundaries were determined by E-cadherin staining, which labels cell membrane, and CD68, which labels cell body. Only those structures with inner cells fully enclosed were counted. Of note, since CIC formation is a dynamic process that mediates the death of internalized cells, which consists of different stages including early stage (viable inner cell) and late stage (dying/dead inner cell), we therefore included those structures with dying inner cells into CIC counts. Quantified CICs were presented as CICs number per tissue core.

### Breast Cancer Subtypes

Breast cancer subtype was judged based on the information on the expression of ER, PR, and Her2 receptors provided along with TMA, where immunohistochemistry (IHC) staining was used to determine ER and PR status, respectively; Her2 status was determined by IHC and FISH. IHC 3^+^ or demonstrating gene amplification by FISH was considered Her2^+^. In this study, we defined breast cancer subtype as: Luminal A with ER and PR > 1%, grade 1/2, Her2^−^, low Ki-67 < 14%; Luminal B (Her2^−^) with ER and/or PR > 1%, grade 3, Her2^−^, high Ki-67 ≥ 14%; Luminal B (Her2^+^) with ER and/or PR > 1%, any grade, Her2^+^, any Ki-67 value; Her2^+^ with Her2^+^ and ER and PR absent; TNBC (triple negative breast cancer) whose tumors were negative for ER, PR, and Her2.

### Statistical Analysis and Nomogram Construction

Overall survival (OS) duration was defined as time from the date of surgery to death or to the most recent contact or visit. Associations between CIC number and the clinicopathological characteristics of the patients were analyzed using the Chi-squared test or Fisher exact test. Survival curves were plotted using the Kaplan–Meier method and the differences in survival curves were compared by the log-rank test. Cox univariate and multivariate regression analysis (22) were conducted to determine the factors that were independently associated with patients' OS. A nomogram was formulated based on the results of multivariate logistic regression analysis and by using the *rms* package of R, version 3.0 (http://www.r-project.org/). The nomogram is based on proportionally converting each regression coefficient in multivariate logistic regression to a 0- to 100-point scale (23). All statistical analyses were performed using SPSS 24.0 software (IBM, Armonk, NY, USA) and GraphPad Prism 6.0. For all these analyses, a *P* < 0.05 was considered statistically significant.

## Results

### Patient Characteristics

In this commercial breast cancer TMA, the study population included 160 patients with breast cancer. All samples were collected from untreated baseline patients. Among the 160 patients, only 148 with complete information were eligible for further analysis because of lost follow-up for five patients and tissue detachment for seven patients during immunostaining. All the patients had a pathological diagnosis of invasive ductal carcinoma (IDC) determined from surgical specimens. Patient characteristics of the 148 patients are summarized in Tables 1A,B. The median age was 51 years (range 29–83 years). Most patients are hormone receptor-positive (70.3%) or Her2^−^ (77.0%). Thirteen patients (8.8%) are in TNM stage I, 91 (61.5%) are in stage II, and 44 (29.7%) are in stage III. The cancer subtypes are distributed as: Luminal A (46.6%), Luminal B (Her2^−^) (10.8%), Luminal B (Her2^+^) (12.8%), HER 2^+^ (10.1%), and TNBC (19.6%).

**Table 1A:** Association of CIC subtypes with clinicopathological characteristics.

**Characteristics**	**oCIC**					**TiT**				**MiT**				**TiM**			
	***N* (%)**	Low^*^n (%)	High^*^n (%)	**χ^2^**	***P***	Low^*^n (%)	High^*^n (%)	**χ^2^**	***P***	**No^#^*n* (%)**	**Yes^#^*n* (%)**	**χ^2^**	***P***	**No^#^*n* (%)**	**Yes^#^*n* (%)**	**χ^2^**	***P***
Total	148	42 (28.4)	106 (71.6)			49 (33.1)	99 (66.9)			122 (82.4)	26 (17.6)			51 (34.5)	97 (55.5)		
Age (years)				0.103	0.749			0.46	0.497			0.045	0.832			2.539	0.111
≤ 60	105 (70.9)	29 (27.6)	76 (72.4)			33 (31.4)	72 (68.6)			87 (82.9)	18 (17.1)			32 (30.5)	73 (69.5)		
>60	43 (29.1)	13 (30.3)	30 (69.7)			16 (37.2)	27 (62.8)			35 (81.4)	8 (18.6)			19 (44.2)	24 (55.8)		
Histological grade				5.823	0.054			4.575	0.102			7.637	**0.022**			2.045	0.36
1	16 (10.8)	8 (50)	8 (50)			8 (50.0)	8 (50.0)			15 (93.8)	1 (6.3)			8 (50.0)	8 (50.0)		
2	127 (85.8)	34 (26.8)	93 (73.2)			41 (32.3)	86 (67.7)			105 (82.7)	22 (17.3)			41 (32.3)	86 (67.7)		
3	5 (3.4)	0 (0.0)	5 (100)			0 (0.0)	5 (100.0)			2 (40.0)	3 (60.0)			2 (40.0)	3 (60.0)		
TNM stage				0.281	0.869			0.052	0.974			0.204	0.903			0.836	0.658
I	13 (8.8)	3 (23.1)	10 (76.9)			4 (30.8)	9 (69.2)			11 (84.6)	2 (15.4)			3 (23.0)	10 (77.0)		
II	91 (61.5)	27 (29.7)	64 (70.3)			30 (33.0)	61 (67.0)			74 (81.3)	17 (18.7)			32 (35.2)	59 (64.8)		
III	44 (29.7)	12 (27.3)	32 (72.7)			15 (34.1)	29 (65.9)			37 (84.1)	7 (15.9)			16 (36.4)	28 (63.6)		
Tumor size				0.210	0.900			0.453	0.797			1.705	0.426			0.647	0.724
<2 cm	35 (23.6)	11 (31.4)	24 (68.6)			13 (37.1)	22 (62.9)			31 (88.6)	4 (11.4)			12 (34.3)	23 (65.7)		
2–5 cm	102 (68.9)	28 (27.5)	74 (72.5)			33 (32.4)	69 (67.6)			83 (81.4)	19 (18.6)			34 (33.3)	68 (66.7)		
>5 cm	11 (7.4)	3 (27.3)	8 (72.7)			3 (27.3)	8 (72.7)			8 (72.7)	3 (27.3)			5 (45.5)	6 (54.5)		
Lymph node metastasis				2.986	0.225			1.8	0.407			0.979	0.613			1.689	0.43
0	62 (41.9)	17 (27.4)	45 (72.6)			20 (32.3)	42 (67.7)			51 (82.3)	11 (17.7)			18 (29.0)	44 (71.0)		
1–2	36 (24.3)	14 (38.9)	22 (61.1)			15 (41.2)	21 (58.3)			28 (77.8)	8 (22.2)			15 (41.7)	21 (58.3)		
> 3	50 (33.8)	11 (22.0)	39 (78.0)			14 (28.0)	36 (72.0)			43 (86)	7 (14)			18 (36.0)	32 (64.0)		
ER status				1.174	0.279			1.619	0.203			0.664	0.415			0.419	0.517
Positive	98 (66.2)	25 (25.5)	73 (74.5)			29 (25.5)	69 (74.5)			79 (80.6)	19 (19.4)			32 (32.7)	66 (67.3)		
Negative	50 (33.8)	17 (34.0)	33 (66.0)			20 (34.0)	30 (66.0)			43 (86.0)	7 (14.0)			19 (38.0)	31 (62.0)		
PR status				1.867	0.172			1.822	0.177			0.567	0.451			0	0.994
Positive	87 (58.8)	21 (24.1)	66 (75.9)			25 (28.7)	62 (71.3)			70 (80.5)	17 (19.5)			30 (34.5)	57 (65.5)		
Negative	61 (41.2)	21 (34.4)	40 (65.6)			24 (39.3)	37 (60.7)			52 (85.2)	9 (14.8)			21 (34.4)	40 (65.6)		
Her2 status				0.343	0.558			0.524	0.469			1.083	0.298			0.498	0.48
Positive	34 (23.0)	11 (32.4)	23 (67.6)			13 (38.2)	21 (61.8)			26 (76.5)	8 (23.5)			10 (29.4)	24 (70.6)		
Negative	114 (77.0)	31 (27.2)	83 (72.8)			36 (31.6)	78 (68.4)			96 (84.2)	18 (15.8)			41 (36.0)	73 (64.0)		
Subtype				5.135	0.274			2.781	0.595			6.532	0.163			3.791	0.435
Lum A	69 (46.6)	21 (30.4)	48 (69.6)			23 (33.3)	46 (66.7)			61 (88.4)	8 (11.6)			25 (36.2)	44 (63.8)		
Lum B (Her2^+^)	16 (10.8)	1 (6.2)	15 (93.8)			3 (18.8)	13 (81.2)			11 (68.8)	5 (31.2)			3 (18.7)	13 (81.3)		
Lum B (Her2^−^)	19 (12.8)	5 (26.3)	14 (73.7)			6 (31.6)	13 (68.4)			13 (68.4)	6 (31.6)			5 (26.3)	14 (73.7)		
Her2^+^/HR^−^	15 (10.1)	6 (40.0)	9 (60.0)			7 (46.7)	8 (53.3)			13 (86.7)	2 (13.3)			5 (33.3)	10 (66.7)		
TNBC	29 (19.6)	9 (31.0)	20 (69.0)			10 (34.5)	19 (65.5)			24 (82.8)	5 (17.2)			13 (44.8)	16 (55.2)		
EGFR status				1.893	0.169			4.253	**0.039**			0.000	1.000			0.093	0.760
Positive	108 (73.0)	34 (31.5)	74 (68.5)			41 (38.0)	67 (62.0)			89 (82.4)	19 (17.6)			38 (35.2)	70 (64.8)		
Negative	40 (27.0)	8 (20.0)	32 (80.0)			8 (20.0)	32 (80.0)			33 (82.5)	7 (17.5)			13 (32.5)	27 (67.5)		
Ki67 status				5.730	**0.017**			2.289	0.130			3.011	0.083			4.409	**0.036**
<14%	106 (71.6)	36 (34.0)	70 (66.0)			39 (36.8)	67 (63.2)			91 (85.8)	15 (14.2)			42 (39.6)	64 (60.4)		
≥14%	42 (28.4)	6 (14.3)	36 (85.7)			10 (23.8)	32 (76.2)			31 (73.8)	11 (26.2)			9 (21.4)	33 (78.6)		

* Low: <15 CICs/core, High: ≥15 CICs/core;

# *No: 0 CICs/core; Yes: ≥1 CICs/core. Lum, luminal. Bold values represent the statistical difference (p <0.05)*.

**Table 1B:** Association of CIC subtypes with clinicopathological characteristics.

	**LiT**				**LiM**				**HeCIC**				
**Characteristics**	***N* (%)**	**No^#^*n* (%)**	**Yes^#^*n* (%)**	**χ^2^**	***P***	**No^#^*n* (%)**	**Yes^#^*n* (%)**	**χ^2^**	***P***	**No^#^*n* (%)**	**Yes^#^*n* (%)**	**χ^2^**	***P***
Total	148	142 (95.9)	6 (4.1)			126 (85.1)	22 (14.9)			38 (25.7)	110 (74.3)		
Age (years)				0.466	0.495			1.762	0.184			1.504	0.220
≤ 60	105 (70.9)	100 (95.2)	5 (4.8)			92 (87.6)	13 (12.4)			24 (22.9)	81 (77.1)		
>60	43 (29.1)	42 (97.7.4)	1 (2.3)			34 (79.1)	9 (20.9)			14 (32.6)	29 (67.4)		
Histological grade				3.949	0.139			0.176	0.916			3.104	0.212
1	16 (10.8)	16 (100.0)	0 (0.0)			14 (87.5)	2 (12.5)			7 (43.8)	9 (56.2)		
2	127 (85.8)	122 (96.1)	5 (3.9)			108 (85.0)	19 (15.0)			30 (23.6)	97 (76.4)		
3	5 (3.4)	4 (80.0)	1 (20.0)			4 (80)	1 (20)			1 (20.0)	4 (80.0)		
TNM stage				1.394	0.498			3.232	0.199			0.499	0.779
I	13 (8.8)	13 (100.0)	0 (0.0)			12 (92.3)	1 (7.7)			3 (23.1)	10 (76.9)		
II	91 (61.5)	86 (94.5)	5 (5.5)			80 (87.9)	11 (12.1)			22 (24.2)	69 (75.8)		
III	44 (29.7)	43 (97.7)	1 (2.3)			34 (77.3)	10 (22.7)			13 (29.5)	31 (70.5)		
Tumor size				0.7825	0.676			2.488	0.288			0.868	0.648
<2 cm	35 (23.6)	34 (97.1)	1 (2.9)			32 (91.4)	3 (8.6)			11 (31.4)	24 (68.6)		
2–5 cm	102 (68.9)	97 (95.1)	5 (4.9)			86 (84.3)	16 (15.7)			24 (23.5)	78 (76.5)		
> 5 cm	11 (7.4)	11 (100.0)	0 (0.0)			8 (72.7)	3 (27.3)			3 (27.3)	8 (72.7)		
Lymph node metastasis				1.609	0.447			2.261	0.323			1.293	0.524
0	62 (41.9)	58 (93.5)	4 (6.5)			53 (85.5)	9 (14.5)			13 (21.0)	49 (79.0)		
1–2	36 (24.3)	35 (97.2)	1 (2.8)			33 (91.7)	3 (8.3)			10 (27.8)	26 (72.2)		
> 3	50 (33.8)	49 (98.0)	1 (2.0)			40 (80.0)	10 (20.0)			15 (30.0)	35 (70.0)		
ER status				0.745	0.391			4.979	**0.026**			0.214	0.644
Positive	98 (66.2)	95 (96.9)	3 (3.1)			88 (89.8)	10 (10.2)			24 (24.5)	74 (75.5)		
Negative	50 (33.8)	47 (94.0)	3 (6.0)			38 (76.0)	12 (24.0)			14 (28.0)	36 (72.0)		
PR status				1.672	0.196			3.408	0.065			0.064	0.800
Positive	87 (58.8)	85 (97.7)	2 (2.3)			78 (89.7)	9 (10.3)			23 (26.4)	64 (73.6)		
Negative	61 (41.2)	57 (93.4)	4 (6.6)			48 (78.7)	13 (21.3)			15 (24.6)	46 (75.4)		
Her2 status				1.865	0.172			0.270	0.603			0.323	0.570
Positive	34 (23.0)	34 (100.0)	0 (0.0)			28 (82.4)	6 (17.6)			10 (29.4)	24 (70.6)		
Negative	114 (77.0)	108 (94.7)	6 (5.3)			98 (86.0)	16 (14.0)			28 (24.6)	86 (75.4)		
Subtype				8.525	0.074			11.268	**0.024**			3.442	0.487
Lum A	69 (46.6)	68 (98.6)	1 (146)			65 (94.2)	4 (5.8)			19 (27.5)	50 (72.5)		
Lum B (Her2^+^)	16 (10.8)	14 (87.5)	2 (12.5)			13 (81.3)	3 (18.7)			2 (12.5)	14 (87.5)		
Lum B (Her2^−^)	19 (12.8)	19 (100.0)	0 (0.0)			15 (78.9)	4 (21.1)			4 (21.1)	15 (78.9)		
Her2^+^/HR^−^	15 (10.1)	15 (100.0)	0 (0.0)			13 (86.7)	2 (13.3)			6 (40.0)	9 (60.0)		
TNBC	29 (19.6)	26 (89.7)	3 (10.3)			20 (69.0)	9 (31.0)			7 (24.1)	22 (75.9)		
EGFR status				2.316	0.128			4.215	**0.040**			0.096	0.757
Positive	108 (73)	102 (94.4)	6 (5.6)			88 (81.5)	20 (18.5)			27 (25.0)	81 (75.0)		
Negative	40 (27)	40 (100.0)	0 (0.0)			38 (95.0)	2 (5.0)			11 (27.5)	29 (72.5)		
Ki67 status				9.292	**0.002**			1.996	0.158			3.987	**0.046**
<14%	106 (71.6)	105 (99.1)	1 (0.9)			93 (87.7)	13 (12.3)			32 (30.2)	74 (69.8)		
≥14%	42 (28.4)	31 (88.1)	11 (11.9)			33 (78.6)	9 (21.4)			6 (14.3)	36 (85.7)		

# *No: 0 CICs/core; Yes, ≥1 CICs/core. Lum: luminal. Bold values represent the statistical difference (p < 0.05)*.

### CIC Subtypes in Breast Cancer Tissues

**Figure 1:**
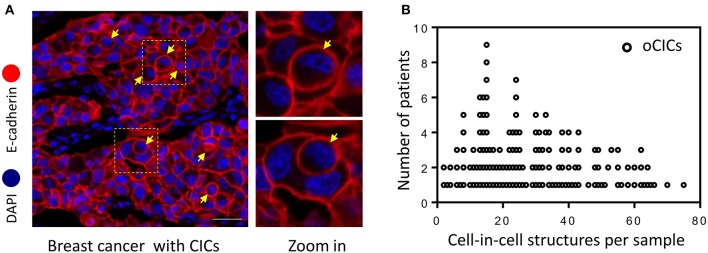
Cell-in-cell structures in breast cancer. **(A)** Representative image for CICs in human breast cancer tissue stained with antibodies for E-cadherin. Nuclei were counterstained with DAPI. Right panel shows zoomed images for boxed region in the left image. Yellow arrows indicate inner cells of cell-in-cell structures. Scale bar: 20 μm. **(B)** Distribution of overall CICs (oCICs) across breast cancer tissues from different patients.

**Figure 2:**
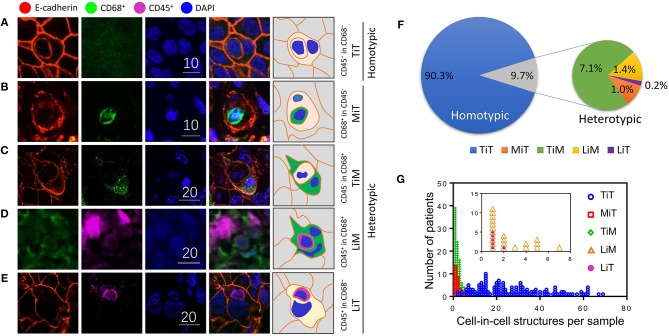
Subtype profiling of cell-in-cell structures in breast cancer. **(A–E)** Representative images for five CIC subtypes as indicated. Right panel of pictures demonstrate the schematic structure for each subtype of CICs. Scale bar: 10 or 20 μm; **(F)** composition analysis of five CIC subtypes; **(G)** distribution of five CIC subtypes across breast cancer tissues from different patients.

Cell-in-cell structures (CICs) were detected in all cancer specimens examined but not in 10 normal breast tissues. The median number of overall CICs (oCICs) in breast cancer tissues is 25 (range 2–75) (Figures 1A,B). A total of five types of CICs were identified in breast cancer tissues (Figures 2A–E). Among them, four subtypes were reported previously by us (9). These four subtypes are designed as (1) TiT (CD45^−^/CD68^−^), for homotypic CICs (hoCICs) formed between tumor cells; (2) LiT (CD45^+^/CD68^−^), for leukocytes inside tumor cells; (3) TiM (CD45^−^/CD68^+^), for tumor cells inside macrophages; and (4) LiM (CD45^+^/CD68^+^), for leukocytes inside macrophages. Notably, a novel subtype was identified as MiT (macrophages inside tumor cell) for the first time in this study. Composition analysis (Figures 2F,G) revealed that hoCIC/TiT (90.3%) constituted majority of the oCICs identified while heterotypic CICs (heCICs) were about 9.7%. Out of the four heCICs, TiM (7.1%) accounts for the majority, and the other three (MiT, LiT, and LiM) account for 1.0, 0.2, and 1.4%, respectively. Accordingly, we chose oCICs, TiT, and TiM for further analysis; MiT was also included for following analysis considering its unique engulfment phenotype, which is opposite to that of TiM.

### Association of CICs With Clinicopathological Characteristics

The relationships between oCICs, TiT, TiM, MiT, and patients' clinical characteristics were investigated and are shown in Table 1. oCIC cutoff was derived from the lower middle of statistical quartile, which turned out to be able to differentiate well-different prognostic groups. High oCICs (≥15) were presented in 106 (71.6%) patients. Low oCICs (<15) were presented in 42 (28.4%) patients. With the same cutoff value of 15 CICs/core, high TiT was observed in 99 (66.9%) patients and low TiT was observed in 49 (33.1%) patients. The oCIC level was positively correlated with Ki-67 expression (χ^2^ = 5.73; *P* = 0.017), whereas the levels of TiT and TiM were positively associated with EGFR expression (χ^2^ = 4.253; *P* = 0.039) and Ki-67 expression (χ^2^ = 4.409; *P* = 0.036), respectively. No significant associations were identified between CICs and other clinical characteristics including TNM stage, HR status, and Her2 status. Interestingly, MiT CICs were almost absent in low-grade cancer tissues, but more frequently present in breast cancer tissues of higher histological grade (χ^2^ = 7.637; *P* = 0.022). This result suggests that MiT may be functionally associated with tumor cell malignancy, as tumor cells “engulfed” the cells (macrophages) that were designed to engulf them.

### Association of CICs With Patient Survival

**Figure 3:**
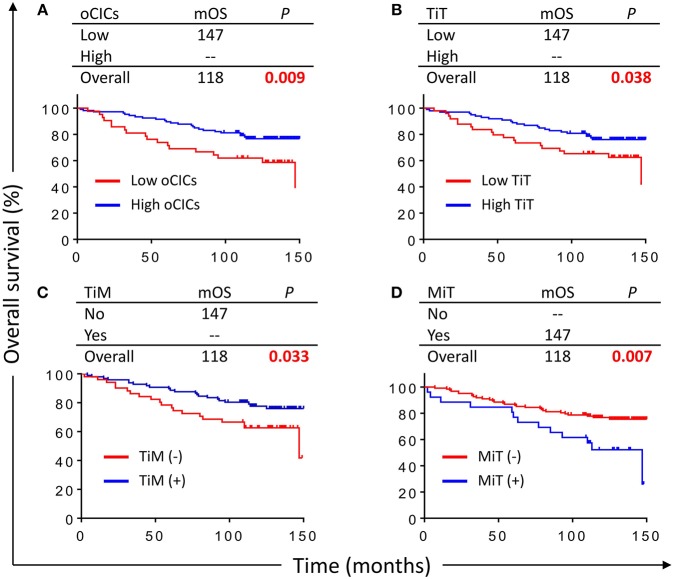
Cell-in-cell structures associate with overall survival (OS) of breast cancer patients. Kaplan–Meier plotting for OS curves of oCICs **(A)**, TiT **(B)**, TiM **(C)**, and MiT **(D)**. High for CICs ≥ 15/core, low for CICs < 15/core; (–) and (+) for cores negative or positive for CICs, respectively.

With a median follow-up period of 118 months (range from 2 to 150 months), 42 (28.4%) patients died. Kaplan–Meier survival curves demonstrated that patients with high oCIC, TiT, or positive in TiM, were associated with favorable OS (*P* = 0.009, 0.038, and 0.033, respectively) as shown in Figures 3A–C. Meanwhile, patients containing MiT displayed shorter median OS (mOS) duration than those without MiT (not reached vs. 147, *P* = 0.007; Figure 3D). LiM and LiT did not show significant correlation with patient survival (data not shown) and therefore were not included into subsequent analysis.

### CICs Selectively Impact Patient Survival of Different Categories

**Figure 4:**
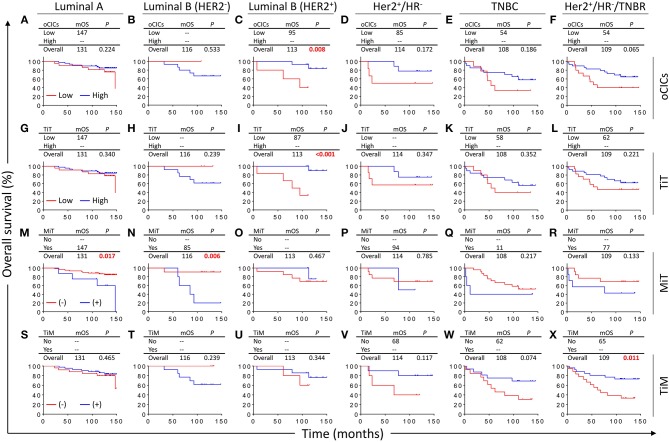
Survival impacts of subtyped cell-in-cell structures on different categories of breast cancers. Kaplan–Meier analyses of prognostic values of oCICs **(A–F)**, TiT **(G–L)**, TiM **(M–R)**, and MiT **(S–X)** in five categories/subtypes of breast cancer. High for CICs ≥ 15/core, low for CICs < 15/core; (–) and (+) for cores negative or positive for CICs, respectively.

Based on the expression levels of receptors (ER, PR, and Her2), breast cancers are routinely classified into five categories/subtypes, which had been shown to affect patient survival (24–27). Nevertheless, patients within each category still show profound differences in OS duration. We therefore further explored the impacts of CICs on patient survival of different categories of breast cancers. Interestingly, CICs and their subtypes tended to preferentially affect the survival of a certain category of breast cancer patients (Figure 4). Low oCICs, at a cutoff of 15 CICs/core, were significantly associated with shorter survival of Luminal B (Her2^+^) patients (mOS: 95 months vs. not reached, *P* = 0.008; Figure 4C), but not other cancer subtypes. Similarly, TiT, which accounts for the majority of oCICs, also selectively impacts Luminal B (Her2^+^) patients with slightly better performance (mOS: 87 months vs. not reached, *P* < 0.001; Figure 4I); The presence of TiM tended to be correlated with the favorable prognosis of patients in HR^−^ group (Her2^+^/HR^−^ and TNBC) (mOS: 65 months vs. not reached, *P* < 0.011; Figure 4X). On the contrary, the presence of MiT was significantly associated with poor patient survival of Luminal A (mOS: 147 months vs. not reached, *P* = 0.017) and Luminal B (Her2^−^) (mOS: 85 months vs. not reached, *P* = 0.006; Figures 4M,N). The selectivity of CIC subtypes on breast cancer subtypes may reflect different cellular compositions and unique intercellular interaction within each subtype of breast cancers.

### CICs Are Independent Prognostic Factors for Breast Cancer

Cox regression test was used to model and assess the relationship between OS and CICs. In univariate analysis, the OS of 148 breast cancer patients was significantly associated with TNM stage (*P* = 0.038), ER (*P* = 0.004), PR (*P* = 0.014), age (*P* = 0.022), cancer subtype (*P* = 0.001), oCIC (*P* = 0.011), TiT (*P* = 0.041), TiM (*P* = 0.036), and MiT (*P* = 0.009; Table 2). In the backward stepwise Cox regressions test, oCICs, TiT, TiM, and MiT served as independent predictors of OS, regardless of age, TNM stage, and cancer subtypes. Patients with high oCICs or TiT had a favorable prognosis with death hazards of 0.423 (*P* = 0.006) and 0.529 (*P* = 0.04), respectively. Similarly, TiM also independently served as a protective factor for breast cancer patients with a death hazard of 0.524 (*P* = 0.037). Conversely, MiT tended to be a risk factor for poor prognosis with a death hazard of 2.608 (*P* = 0.05), which is higher than that of TNM stage (HR = 2.117, *P* = 0.01) and that of cancer subtypes (HR = 1.310, *P* = 0.006; Table 3).

**Table 2:** Association of overall survival with clinicopathological parameters and CICs by univariate Cox-regression analysis.

**Characteristics**	**HR (SE)**	**95% CI**	***P***
Age	2.050 (0.313)	1.110–3.787	**0.022**
Histological grade	1.894 (0.638)	0.777–4.616	0.160
TNM stage	1.762 (0.272)	1.033–3.004	**0.038**
Tumor size	1.402 (0.338)	0.765–2.568	0.274
Lymph node metastasis	1.072 (0.318)	0.757–1.517	0.697
ER status	0.409 (0.310)	0.223–0.749	**0.004**
PR status	0.462 (0.312)	0.250–0.853	**0.014**
HER2 status	1.093 (0.363)	0.537–2.224	0.807
Cancer subtype	1.360 (0.094)	1.130–1.636	**0.001**
EGFR status	1.700 (0.393)	0.786–3.674	0.177
Ki67 status	1.012 (0.342)	0.518–1.977	0.973
oCIC	0.453 (0.312)	0.245–0.835	**0.011**
TiT	0.531 (0.310)	0.289–0.975	**0.041**
MiT	2.409 (0.334)	1.251–4.639	**0.009**
TiM	0.523 (0.309)	0.285–0.958	**0.036**

*Bold values represent the statistical difference (p < 0.05)*.

**Table 3:** Multivariate Cox-regression analysis of overall survival with CICs as a variable.

**Variables**	**oCIC**			**TiT**			**MiT**			**TiM**		
	***n***	**HR**	***P***	***n***	**HR**	***P***	***n***	**HR**	***P***	***n***	**HR**	***P***
CICs		0.423 (0.227–0.785)	**0.006**		0.529 (0.288–0.973)	**0.04**		2.608 (1.344–5.063)	0.05		0.524 (0.286–0.962)	**0.037**
≤ 15	42			49			122^*^			49^*^		
>15	106			99			26^#^			99^#^		
Age		2.038 (1.055–3.937)	**0.034**		–	–		2.173 (1.138–4.147)	**0.019**		–	–
≤ 60	105			105			105			105		
>60	43			43			43			43		
TNM stage		2.119 (1.189–3.774)	**0.011**		1.692 (1.001–2.860)	**0.049**		2.117 (1.192–3.759)	**0.01**		–	–
I	13			13			13			13		
II	91			91			91			91		
III	44			44			44			44		
Subtype		1.307 (1.084–1.576)	**0.005**		1.350 (1.121–1.625)	**0.002**		1.310 (1.081–1.588)	**0.006**		1.354 (1.128–1.626)	**0.001**
Lum A	69			69			69			69		
Lum B (Her2^+^)	16			16			16			16		
Lum B (Her2^−^)	19			19			19			19		
Her2^+^/HR^−^	15			15			15			15		
TNBC	29			29			29			29		

* Number of samples negative in MiT or TiM;

# *number of samples positive in MiT or TiM; Lum, luminal. Bold values represent the statistical difference (p < 0.05)*.

### CICs Profoundly Contribute to Predict Patient Outcomes

**Figure 5:**
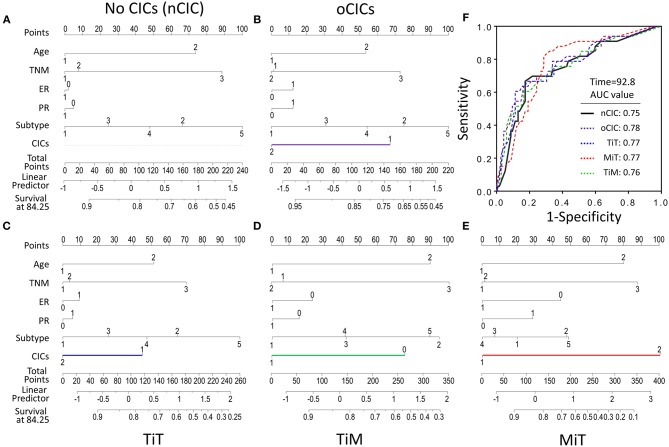
Contribution of subtyped cell-in-cell structures to survival prediction by nomogram analysis. **(A)** Prognostic nomogram in the absence of CICs as a viable. **(B–E)** Prognostic nomogram with oCICs **(B)**, or TiT **(C)**, or TiM **(D)**, or MiT **(E)** as a variable, respectively. For the probability of patient survival for 84.25 months, summing up the points from individual variable, locating its position on the axis of total points to determine the corresponding survival probability based on the bottom line (survival at 84.25) of the nomogram. In addition to CICs, age, TNM stage, ER and PR statuses, and cancer subtypes were included in the nomogram analysis. **(F)** The AUC of different nomogram models from observed data.

To evaluate the contribution of CICs and their subtypes to predict patient prognosis, we constructed nomograms incorporating each type of CICs together with other independent prognostic factors (age, TNM stage, and cancer subtype) and hormone receptors (ER and PR). As shown in Figure 5, the contributions of oCICs, TiT, and TiM are comparable to those of TNM stage or cancer subtype (Figures 5B–D). Moreover, MiT even dominates over all the other factors in the contribution of predicting patient survival (Figure 5E). Each subtype of these variables was assigned a score on the point scale, and by locating the total score from all variables on the total point scale, the probability of patient survival could be estimated at the time point of 84.25 months. Actually, incorporating CICs indeed improved the prediction performance though slightly [area under curve (AUC) from 0.75 to 0.78] (Figure 5F).

## Discussion

Although CICs had been extensively reported in various human tumors for a century (10), this study represents the first systemic analysis of CIC subtypes in human cancers. Our results suggest that subtype-based prognostic analysis is informative and essential as heterogeneous tumors generally give rise to different types of CICs that could impact patient outcomes differently or even oppositely. The different roles that each CIC subtype has in patient prognosis reflect their intrinsic differences in biological functions. For example, the “competitive engulfment” between tumor cells and macrophages generates two types of CICs: MiT and TiM. The presence of MiT CICs for engulfment of macrophage by tumor cells, which was identified for the first time, may surrogate gain of malignant function and successful immune evasion by tumor cells, and therefore is associated with poor patient survival in breast cancer in this work. Meanwhile, formation of TiM CICs, from engulfment of tumor cells by macrophages, suggests active elimination of tumor cells by the first-line innate immune system, and therefore is correlated with favorable prognosis. In agreement with which, activating macrophages by antibodies targeting CD47-SIRP interaction could effectively inhibit tumor growth in both animal experiments and clinical trials (28–30). Hence, it is conceivable that TiM, and probably MiT as well, may serve as good candidates of biomarker for predicting cancer therapy efficacy, which warrants further investigation.

To our surprise, CICs and their subtypes, as functional prognostic factors as proposed above, not only show prognostic power comparable to those of traditional pathological parameters such as TNM stage and cancer subtyping, but also even superior in the case of MiT subtype as shown in nomogram analysis in Figure 5. This result suggests that functional pathology assisted by CIC scoring might be clinically beneficial for patient diagnosis, and CICs hold promising potential as novel pathological index functionally complementary to existing parameters. The advantage of CICs over traditional parameters lies in that it is not simply quantification of certain types of cells or molecules; instead, CICs contain implanted information of functional interactions among heterogeneous cells, which are integrated output of complicated signaling networks. Actually, CICs and their subtypes demonstrated even more values in subtyped breast cancer as shown in Figure 4. Of note, the presence of MiT was significantly associated with poor prognosis of HR^+^/Her2^−^ group patients, which includes Luminal A and Luminal B (Her2^−^) and stands for the largest population of breast cancer patients (>70%). When performing statistical analysis in this population (Table 4), TNM stage, histological grade, tumor size, and TiM are all adversely associated with patient survival (*P* = 0.077, 0.005, 0.020, and <0.001, respectively) in univariate analysis, whereas only tumor size (HR = 4.678, *P* = 0.014) and MiT (HR = 5.854, *P* < 0.001) independently predict patient survival in multivariate analysis. In both analysis, MiT displays the best performance over the others (Table 4). Based on this observation, we propose MiT as an independent adverse prognostic factor for breast cancer (particularly valuable for HR^+^/Her2^−^ group), which may serve as a more general biomarker for other type of cancers, such as pancreatic cancer as reported in the co-submitted work. Of course, for either cases, further validations in larger cohort of patients are warranted.

**Table 4:** Univariate and multivariate cox-regression analysis of prognostic parameters in HR^+^/HER2^−^ breast cancer patients.

**Variables**	**Univariate**			**Multivariate**		
**HR^**+**^/HER2^**−**^**	**HR**	**95% CI**	***P***	**HR**	**95% CI**	***P***
TNM stage	2.144	0.920–4.993	0.077	–	–	–
Histological grade	4.718	1.598–13.928	**0.005**	–	–	–
Tumor size	3.483	1.222–9.929	**0.020**	4.678	1.369-15.982	**0.014**
MiT	5.309	2.076-13.579	**<** **0.001**	5.854	2.239-15.310	**<** **0.001**

*Bold values represent the statistical difference (p < 0.05)*.

Interestingly, our work identified oCICs and TiT/hoCICs as favorable factors for the prognosis of breast cancer, which likely disagrees with the traditional notion that CICs were correlated with tumor malignancy (19, 31) and predicted adverse patient outcomes as reported in other types of cancer such as head and neck cancer (32), This may be attributed to different cancer type and stages. Actually, despite Abodief et al. reporting that CICs were associated with a higher histological grade in a relatively small number (*n* = 50) of breast cancer (21), to our best knowledge, this study is the first and largest (*n* = 148) systemic prognostic analysis for CICs in human breast cancer. In fact, when revisiting our data, the oCICs, TiT, and MiT were also more frequent in the higher-grade breast cancer (*P* = 0.054, 0.102, and 0.022, respectively); therefore, a positive correlation with higher histological grade does not necessarily translate into adverse prognosis, it may also stand for a protective response to uncontrolled tumor cell growth. Our experimental data were consistent with this phenotype, where enforced activation of entotic CIC formation in breast cancer cells effectively suppresses anchorage-independent tumor growth while inhibiting CIC formation (11, 33). In addition, the tumor stage may be another factor affecting CIC formation and effects. As identified previously, CIC formation was a clonal selection process that mediates competition within heterogeneous tumor cells (34) and occurred at early and middle stages of tumor development (35, 36), though this selection eventually resulted in survival of malignant clones at the expense of relative benign tumor clones, the net outcomes were the retarded tumor growth at these stages. The tumor will enter the late stage of rapid uncontrolled growth once CIC formation was inactivated (8, 35). Consistently, most of the patients are in relative early stages (Table 1).

The impacts of the present study were limited by several factors. For example, among 148 patients enrolled for analysis, <1/3 of the patients had died with endpoint for OS. Therefore, median OS was not reached in some groups by Kaplan–Meier plotting. This is largely attributed to a good prognosis of operable early breast cancer, and a study with prolonged follow-up will be helpful in the future. Second, commercial TMA was used to explore the prognostic value of CICs in this study, and some information, such as subsequent treatment following surgery and date on first relapse, was not available, which prevents us from evaluating the effect of various treatment on patient survival and disease-free survival (DFS) as well. These considerations warrant further investigation in the future.

In summary, this study reported the first subtype-based CIC profiling in human breast cancer and identified CICs and their subtypes (TiT, TiM, and MiT) as valuable prognostic markers to predict patient survival in specified population. Particularly interesting is that MiT, formed by internalization of macrophages into tumor cells, was identified as a potent adverse prognostic marker, which appears to work across different types of human cancers. We also propose that functional pathology, which incorporates functional indexes like CIC scoring into traditional pathology, is promising in enhancing clinical diagnosis and guiding cancer therapy.

## Data Availability Statement

The raw data supporting the conclusions of this manuscript will be made available by the authors, without undue reservation, to any qualified researcher.

## Ethics Statement

The TMA was purchased from Shanghai Outdo Biotech Co. Ltd., who had collected the tumor samples under the highest ethical standards with the donor being informed completely and with their consent in accordance with the Declaration of Helsinki. The Outdo Biotech Co. Ltd. was part of National Human Genetic Resources Sharing Service Platform and approved to provide TMA with license No.: 2005DKA21300.

## Author Contributions

QS and HH: concept and design. ZN, JF, and BZ: staining and imaging. XZ, HQ, MW, LG, and YZ: data acquisition. QS, HH, ZC, and XZ: manuscript drafting. YT: pathological judgment and confirmation. XZ and HH: statistical analysis. QS, HH, MY, ZC, and YT: funding.

### Conflict of Interest

The authors declare that the research was conducted in the absence of any commercial or financial relationships that could be construed as a potential conflict of interest.
